# Global dynamics of SARS-CoV-2 clades and their relation to COVID-19 epidemiology

**DOI:** 10.1038/s41598-021-87713-x

**Published:** 2021-04-19

**Authors:** Samira M. Hamed, Walid F. Elkhatib, Ahmed S. Khairalla, Ayman M. Noreddin

**Affiliations:** 1 Department of Microbiology and Immunology, Faculty of Pharmacy, October University for Modern Sciences and Arts (MSA), 6th of October, Giza, 12451 Egypt; 2 Microbiology and Immunology Department, Faculty of Pharmacy, Ain Shams University, African Union Organization St, Abbassia, 11566 Cairo Egypt; 3 Department of Microbiology & Immunology, Faculty of Pharmacy, Galala University, New Galala city, Suez, Egypt; 4 Microbiology and Immunology Department, Faculty of Pharmacy, Beni-Suef University, Beni-Suef, 62511 Egypt; 5 Department of Biology, University of Regina, Regina, SK Canada; 6 Department of Biology, Coast Mountain College, British Columbia, Canada; 7 Department of Pharmacy Practice and Clinical Pharmacy, Faculty of Pharmacy, Galala University, New Galala city, Suez, Egypt; 8 Department of Internal Medicine, School of Medicine, University of California Irvine, Irvine, CA 92697 USA

**Keywords:** Infectious diseases, Epidemiology, Evolutionary developmental biology

## Abstract

Expansion of COVID-19 worldwide increases interest in unraveling genomic variations of novel SARS-CoV-2 virus. Metadata of 408,493 SARS-CoV-2 genomes submitted to GISAID database were analyzed with respect to genomic clades and their geographic, age, and gender distributions. Of the currently known SARS-CoV-2 clades, clade GR was the most prevalent worldwide followed by GV then GH. Chronological analysis revealed expansion in SARS-CoV-2 clades carrying D614G mutations with the predominance of the newest clade, GV, in the last three months. D614G clades prevail in countries with more COVID-19 cases. Of them, the clades GH and GR were more frequently recovered from severe or deceased COVID-19 cases. In contrast, G and GV clades showed a significantly higher prevalence among asymptomatic patients or those with mild disease. Metadata analysis showed higher (p < 0.05) prevalence of severe/deceased cases among males than females and predominance of GR clade in female patients. Furthermore, severe disease/death was more prevalent (p < 0.05) in elderly than in adults/children. Higher prevalence of the GV clade in children compared to other age groups was also evident. These findings uniquely provide a statistical evidence on the adaptation-driven evolution of SARS-CoV-2 leading to altered infectivity, virulence, and mortality.

## Introduction

Late in December 2019, an outbreak of atypical pneumonia of unknown etiology was described in Wuhan province in China. A novel coronavirus named “Severe Acute Respiratory Syndrome CoronaVirus 2 (SARS-CoV-2)” was then identified as the etiologic agent^1,2^. Later, the disease was designated COrona VIrus Disease-2019 (COVID-19)^3^. The rapid expansion of COVID-19 cases in number and geographic distribution prompted the World Health Organization (WHO) to declare a global health emergency. Containment of the disease was hindered by the lack of antiviral treatment, lack of vaccines and existence of asymptomatic carriers. On March 11, 2020, COVID-19 was officially classified by the WHO as a pandemic.

After declaration of COVID-19 as pandemic, there was a global interest in exploring genomic variations in the novel virus. The first genomic sequence of SARS-CoV-2 was reported by Wu and colleagues^2^. Subsequently, publicly available resources were developed to provide dynamic and updated data on SARS-CoV-2 genome, thus offering an extraordinary opportunity for comparative genomic studies. Among the open access repositories of SARS-Cov-2 genomic sequences are the Global Initiative for Sharing All Influenza Data (GISAID) database (https://www.gisaid.org)^4^, National Center for Biotechnology Information database (NCBI) (www.ncbi.nlm.nih.gov), and Virus Pathogen Resource database (ViPR) (www.viprbrc.org). Genome analysis tools were also provided by several platforms such as The China National Center for Bioinformation (https://bigd.big.ac.cn/ncov/tool/annotation)^5^ Nextstrain project (https://nextstrain.org)^6^, and CoV-GLUE (http://cov-glue.cvr.gla.ac.uk)^7^.

According to GISAID nomenclature system, most of the currently sequenced SARS-CoV-2 genomes were clustered into one of seven major clades. Such clades include L, to which SARS-CoV-2 virus reference strain belongs, S, V, G, GH, GR, and GV. They exhibit few changes in relation to the reference strain (GenBank accession number NC_045512, GISIAD accession ID: EPI_ISL_402124). Such changes include: L84S in NS8 for clade S; coexisting L37F and G251V mutations in NSP6 and NS3, respectively for clade V; D614G mutation in the spike protein (S) for clade G. In addition to D614G, NS3-Q57H, N-G204R and S-A222V mutations characterize the clades GH, GR and GV, respectively. Genomes that don’t belong to any of the seven major clades had the designation “O clade”.

Given that most of the immune-based therapeutics and diagnostics of COVID-19 are based on the protein sequence of Wuhan reference strain spike^8^, their efficacy could potentially be affected by genomic variations and the associated altered viral phenotype. Moreover, the influence of genetic mutations on the infectivity and/or virulence of SARS-CoV-2 is yet to be established^9^. The acquisition of mutations imparting higher infectivity, virulence and/or immunological resistance is thus an eminent threat. Accordingly, active genomic surveillance and close monitoring of the genomic sequence dynamics of SARS-CoV-2 is urgently required to: (a) trace the pattern of geographic spread of the virus during the ongoing pandemic^10,11^; (b) ensure the effectiveness of vaccines and immune-based diagnostic or therapeutic interventions currently in use or under investigation^9^; and (c) identify putative therapeutic targets^12–14^.

Geographic, gender, and age discrepancy of COVID-19 disease outcome have been reported by several studies^15–18^. Whether this correlates to SARS-CoV-2 genomic variation is still unclear. In addition to laboratory investigations, statistical approaches correlating the distribution of viral clades in different groups to disease severity might provide a good evidence on this bias. The current study aims to analyze the geographic, gender and age distribution of SARS-CoV-2 genomic clades with respect to COVID-19 disease epidemiology.

## Results

### Geographic distribution of SARS-CoV-2 clades

As of January 25, 2021, WHO reported a total number of 98,925,221 confirmed COVID-19 cases and 2,127,294 deaths^19^. The calculated world case fatality rate (CFR) was 2.15%. Of all continents, the highest number of COVID-19 cases was reported from North America while most deaths were in Europe. South America had the highest calculated CFR.

GR was the most common clade (34.0%), followed by GV (22.3%) and GH (21.4%). Lower prevalence was noted for their parent clade, G, (15.8%). Other less common clades including L, S, and V were identified in 1.2%, 2.1% and 1.5% of the submitted genomes, respectively. About 1.7% of the genomes were not clustered into any of the major clades and thus had the designation “clade O”.

**Figure 1:**
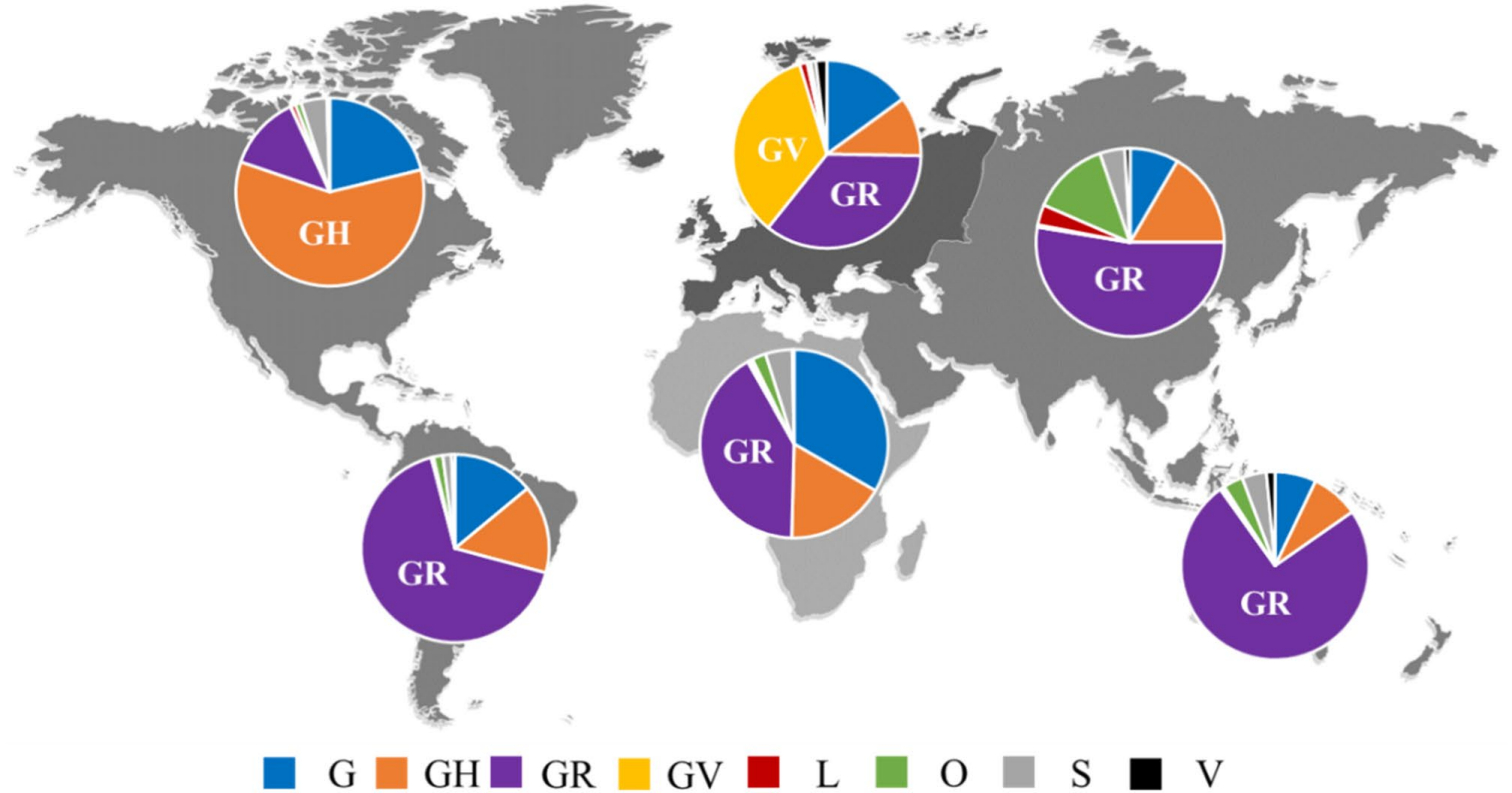
Continent distribution of various SARS-CoV-2 clades. The figure shows the predominance of clade GR in Africa, Asia, Oceania, and South America and the predominance of clade GH in North America. The clades GR and GV predominated in Europe.

Analysis of the continent distribution of SARS-CoV-2 clades (Fig. 1) showed that clade GR was the most frequently identified among the genomes submitted from four continents namely Africa (41.1%), Asia (52.7%), Oceania (74.8%), and South America (66.8%). Clade GH was the most common in North America (59.0%). In Europe, both GR (35.5%) and the newly emerged clade, GV (34.6%) predominated.

The number of coexisting clades was compared between countries with respect to different disease epidemiology parameters including the number of cases, total number of deaths and CFRs. Viral strains belonging to all known clades coexisted in 31 countries (21.1%). Of them, 61.3% reported above median local values for the studied disease epidemiology parameters.

Mann–Whitney test showed a significant difference in the distribution of the number of coexisting clades in the two groups with respect to the total number of cases (P-value < 0.001), total deaths (P-value < 0.001) and CFRs (P-value = 0.020). Higher medians of the number of coexisting clades were shown in the group of countries where above median cases, deaths and CFRs were recorded.

The impact of the distribution of individual clades on the disease epidemiology was also analyzed. Distribution bias of some clades was noted, as shown in Table 1. This was statistically significant for all clades with all disease epidemiology parameters.

**Table 1:** Geographical distribution of SARS-CoV-2 clades with respect to disease epidemiology parameters.

Geographic region	L	S	V	G	GH	GR	GV
Countries showing above median number of cases	1.2%	1.9%	1.5%	16.1%	21.9%	32.8%	23.5%
Countries showing below median number of cases	2.1%	4.4%	1.7%	12.7%	16.1%	45.8%	10.4%
Countries showing above median number of deaths	1.3%	1.9%	1.5%	16.1%	21.9%	32.6%	23.5%
Countries showing below median number of deaths	1.0%	4.4%	1.7%	12.2%	16.1%	47.5%	10.3%
Countries showing above median CFRs	1.3%	2.1%	1.7%	15.4%	22.2%	34.3%	21.8%
Countries showing below median CFRs	1.2%	2.1%	0.5%	17.7%	17.3%	32.5%	24.7%

Chi-square test showed that the distribution bias of all clades among the groups of countries showing above median and below median values for all disease epidemiology parameters was statistically significant (P-value < 0.05).

Among all studied cases, patient’s clinical status were specified for only 2,634. Based on the provided data, such cases were grouped into asymptomatic/mild cases and severe/deceased cases. Although clades GH, and GR were significantly more prevalent among viral genomes isolated from severe/deceased cases, L clade showed the same distribution but with P-value > 0.05. In contrast all other clades showed higher prevalence in asymptomatic/mild cases than severe/deceased ones. This was statistically significant for all clades except clade V (Table 2).

**Table 2:** Distribution of SARS-CoV-2 clades with respect to patient’s clinical status.

Patient status	L	S	V	G	GH	GR	GV
Asymptomatic/mild (n = 1,520)	0.5%	17.8%	0.6%	24.7%	13.9%	26.7%	5.7%
Severe/deceased (n = 1,114)	1.1%	2.2%	0.4%	19.7%	31.4%	36.9%	0.2%
P-value	0.108	< 0.001*	0.617	0.002*	< 0.001*	< 0.001*	< 0.001*

P-values were calculated using Chi-square test. *P-values < 0.05 are statistically significant.

**Figure 2:**
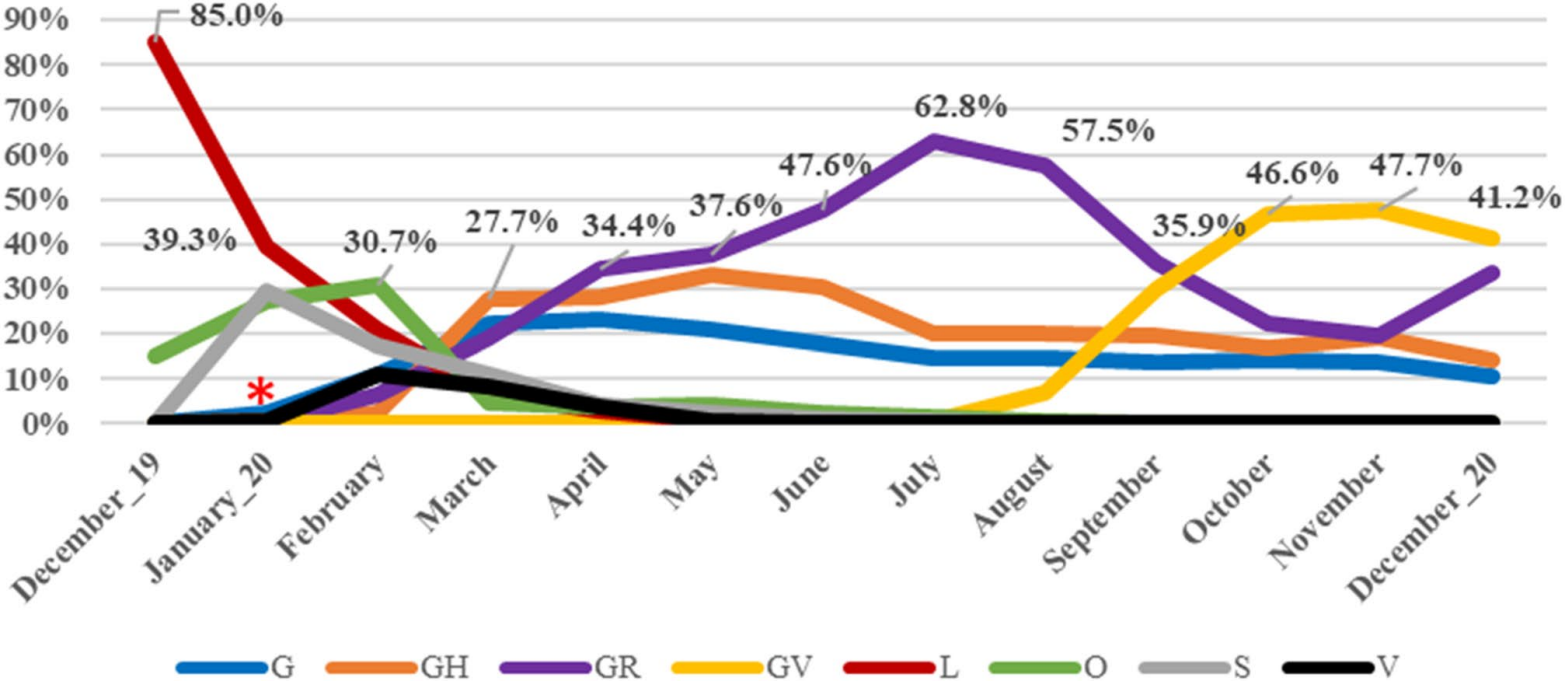
Global chronological distribution of SARS-CoV-2 clades in the period from December 2019 till December 2020. Percentages are used for labelling the predominant clade in each month. The emergence of D614G mutation is marked by a red asterisk.

Analysis based on the chronological distribution of SARS-CoV-2 clades was done for 404,496 cases for which the exact date of collection was available. The analysis showed that clade L predominated at the beginning of the pandemic. Thereafter, new clades evolved including the clades S and V. Viral clades carrying D614G mutation also emerged. Of them, clade G first emerged and soon split into the clades GH and GR. A gradual regression of all clades was then noted with an expansion of clade GR that predominated the scene for six months till the emergence of the last clade, “GV”, by which it was rapidly outweighed. Currently, GV clade is distributed in at least 49 countries predominantly in the United Kingdom (73.3% of the reported GV cases). The global chronological distribution of SARS-CoV-2 clades is shown in Fig. 2. The origins and the evolution time of SARS-CoV-2 clades inferred from the genomes submitted to GISAID are shown in Table 3.

**Table 3:** The origins and the evolution time of SARS-CoV-2 clades.

Clades	Origin	Date of first isolation	GISAID accession ID of first genome
Clade L	Asia/China	December 24, 2020	EPI_ISL_402123
Clade S	Asia/China	January, 2020	EPI_ISL_413691, EPI_ISL_413695
Clade V	Asia/China	January 23, 2020	EPI_ISL_421223
Clade G	Europe/Germany	January 1, 2020	EPI_ISL_450201, EPI_ISL_450202, EPI_ISL_450205, EPI_ISL_450206
Clade GH	North America/United States of America	January 3, 2020	EPI_ISL_861025
Clade GR	Europe/Czech Republic	January 6, 2020	EPI_ISL_850687
Clade GV	Europe/United Kingdom	March 7, 2020	EPI_ISL_724371

### Gender distribution of SARS-CoV-2 clades

Analysis of 96,350 cases (Males = 49,454, Females = 46,896) for which patient gender was specified showed gender distribution bias for some clades (Table 4). This was statistically significantly for clades L and G that showed higher prevalence in males than females. Similarly, the clades GR and GV were more frequently isolated from females than males with P-value less than 0.05.

**Table 4:** Gender Distribution of SARS-CoV-2 clades.

Gender	SARS-CoV-2 clades						
	L	S	V	G	GH	GR	GV
Females (n = 46,896)	1.6%	3.6%	2.7%	18.8%	24.8%	36.8%	9.1%
Male (n = 49,454)	2.1%	3.8%	2.6%	19.1%	25.8%	33.3%	8.5%
P-value	< 0.001*	0.106	0.545	0.025*	0.201	< 0.001*	0.001*

P-values were calculated using Chi-square test. *P-values < 0.05 are statistically significant.

**Figure 3:**
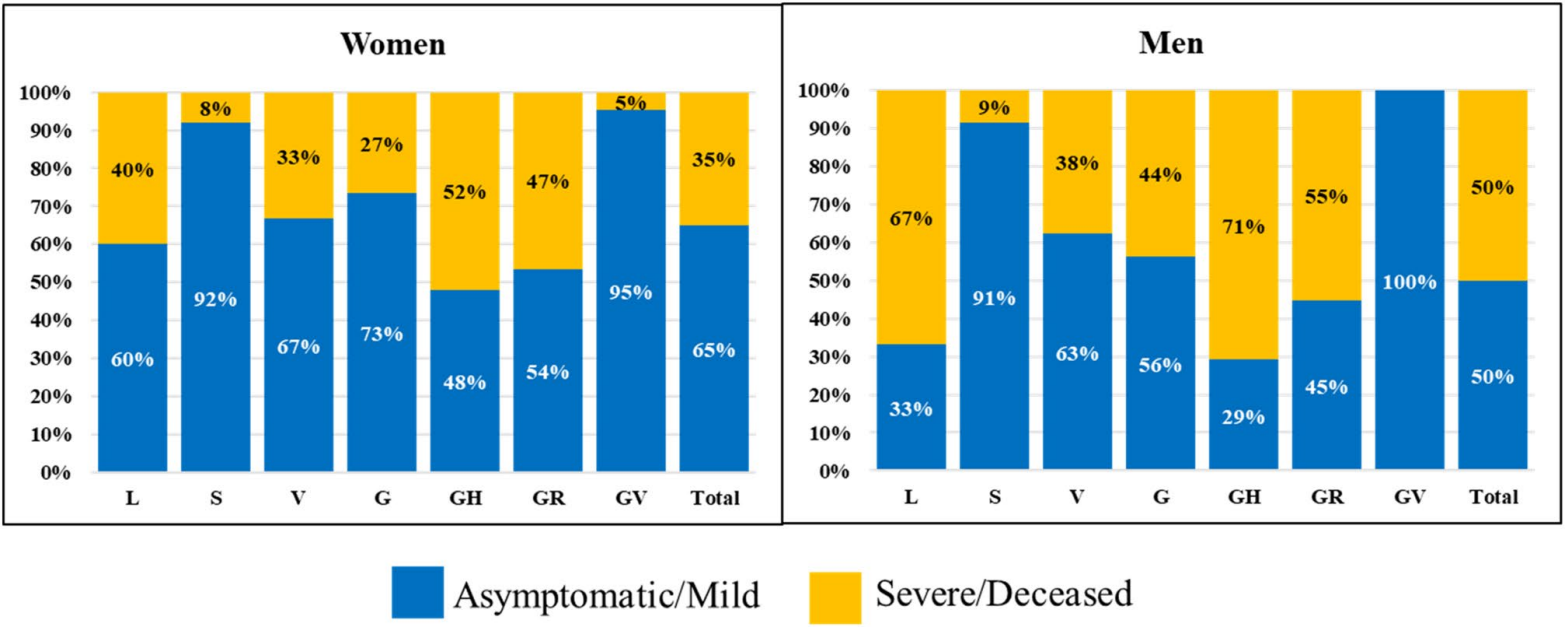
Clinical status of patients infected by SARS-CoV-2 of different clades in different gender groups.

The severity of cases in both genders were compared in 2495 cases for which both gender and patient’s clinical status are known. Among the group of cases for which the clinical status was recorded as severe or deceased, the number of male patients was significantly higher than female patients (49.9% versus 35.4%, P-value < 0.001). Clinical status of patients infected by SARS-CoV-2 of different clades in different gender groups is shown in Fig. 3.

### Age distribution of SARS-CoV-2 clades

**Figure 4:**
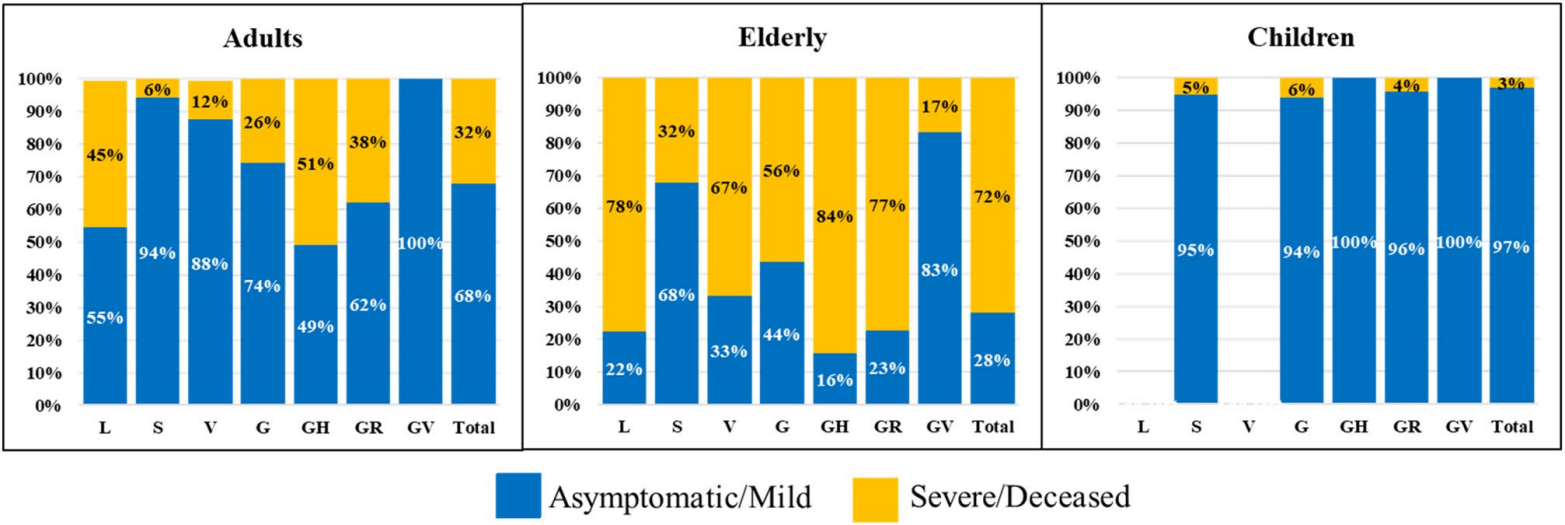
Clinical status of patients infected by SARS-CoV-2 of different clades in different age groups.

The distribution of the genomes that belonged to different clades in different age groups was analyzed among 95,848 cases for which the patient age was specified (Table 5). As shown in Table 5, viral isolates belonging to clade GR were more common in adult patients than other groups. Meanwhile, children were the age group from which the clades GH and GV were more frequently isolated. Isolation of all other clades was commonest in elderly patients compared to others. Clinical status of patients of different age groups from which viral genomes belonging to different clades were isolated is shown in Fig. 4.

**Table 5:** Age groups distribution of SARS-CoV-2 clades.

Age groups	SARS CoV-2 clades						
	L	S	V	G	GH	GR	GV
Adults (n = 68,704)	1.7%	3.5%	2.0%	17.9%	25.4%	36.8%	8.1%
Children (n = 5431)	0.8%	2.3%	1.3%	17.0%	26.4%	31.7%	17.9%
Elderly (n = 21,713)	2.4%	4.7%	4.7%	20.4%	23.4%	32.1%	9.4%

A significant correlation was found between age groups and patient’s clinical status. The analysis included 2,524 cases for which both patient age and clinical status are known (Fig. 4). Severe/deceased cases were significantly more prevalent in elderly than in adults (71.9% vs 31.6%, Pearson Chi-Square P-value < 0.001) or in children (71.9% vs 3.4%, Pearson Chi-Square P-value < 0.001). They were also more frequently reported among adults compared to children in a statistically significant manner (31.6 vs 3.4%, Fisher’s Exact test P-value < 0.001).

## Discussion

A relatively higher genomic stability was reported for SARS-CoV-2 compared to SARS-CoV^20^. Nevertheless, SARS-CoV-2 genomes sequenced so far were clustered into at least seven major clades, as defined by GISAID database. Whether the genetic variability in SARS-CoV-2 clades arises due to an ongoing adaptation or merely due to genetic drift is still unknown. Lack of distinct evolutionary patterns or signatures in SARS-CoV-2 genomes was reported^21^, while independently emerged recurrent mutations were also identified^22^, suggesting an ongoing adaptation. Whether this possible adaptation provides more fitness for transmission and/or virulence is a matter of concern. In the current study, the metadata of 408,493 SARS-CoV-2 genomes submitted to GISAID EpiCoV database as of January 25, 2021 were analyzed with respect to genomic clades and their geographic, age, and gender distribution.

Most of the genomes belonged to one of seven major clades namely L, S, V, G, GH, GR or GV. In addition, genomes that were not clustered to any of the seven major clades (clade O) were also identified. About 93.5% of the genomes belonged to the clades with D614G mutation including the clades G, GH, GR and GV. Of them Clade GR was the most frequently identified followed by GV and GH. Earlier in January, clade G characterized by spike D614G mutation was identified and rapidly predominated the pandemic. The mutation was found to be located in a heavily glycosylated residue in the viral spike that is highly conserved in this species^23^. Theoretical evidence strongly suggests that mutations in this region could be coupled to altered capacity for host cell membrane fusion^23–25^, an effect that should also lead to higher person to person transmission and pathogenicity. An experimental evidence was later provided by Korber and colleagues^9^, who could link this mutation to greater infectivity and higher viral loads in COVID-19 patients. Sub-clusters of clade G then started to evolve including the clades GH, GR and more recently clade GV. The analysis of the chronological distribution of SARS-COV-2 clades in the current study showed that there was much expansion in the number of sequenced genomes that were clustered into the GR clade compared to clade G. A regression in the number of genomes clustered into clade GH was also evident. The newly introduced clade, GV, could also outweigh clade GR in the last few months suggesting higher fitness for transmission by the newer clades compared to their ancestral one. Based on the mentioned facts, the hypothesis of an adaptation-driven genetic evolution is stronger. However, an experimental evidence, providing comparison between clades, is yet to be established.

Adequate scientific elucidation of the reasons behind the rapid transmission and higher mortality rates of COVID-19 in some geographic regions compared to others is still demanding. Apart from public health issues, intrinsic factors related to viral genome may be implicated. Whether the geographic distribution bias of SARS-CoV-2 clades is related to the discrepancy of COVID-19 disease severity observed worldwide is still unclear^26^. In agreement with others^21,27^, a geographic distribution bias of SARS-CoV-2 clades was evident in the current analysis. The predominance of certain clades in different continents with respect to local disease epidemiology parameters was also analyzed. GH clade predominated North America were the highest number of cases was reported while GR predominated South America, the top ranked continent with respect to CFR. Both GR and GV were equally prevalent in Europe from which most deaths were reported. Coexistence of all clades was evident in 21.1% of the contributing countries accompanied, in most cases, by relatively higher COVID-19 cases, deaths and CFRs.

Tracking the distribution of individual clades in different countries with respect to disease epidemiology parameters showed higher prevalence of all clades with D614G mutation among the group of countries that showed above median total number of cases than others. With respect to the case fatality rates, only the clades G and GV were more frequently identified among the genomes submitted from the group of countries showing below median CFRs. Such findings suggest higher transmission of viral strains whose genome belong to all clades with D614G mutation. Higher virulence of clades GH and GR compared to G and GV is also suspected. To further examine this hypothesis, the distribution of all clades among viral genomes from patients with asymptomatic or mild disease and those from severe disease or deceased patients was analyzed. The clades GH and GR significantly showed higher prevalence among the group of severe disease or deceased patients. This is in line with the previous finding of higher viral loads in patients infected by SARS-CoV-2 virus strains harboring D614G genomic mutations^9^. In addition, lower prevalence of the clades S, G and GV among severe or deceased cases was also statistically significant. In agreement with this finding, clade S was also found to be less prevalent among the group of countries that showed above median values for the studied epidemiologic parameters. Although the reference strain of SARS-CoV-2 belonged to the L clade that also had higher prevalence at the beginning of the pandemic, clade S was found to be evolutionarily more related to animal coronaviruses^28^. In agreement with our findings, this suggests higher fitness for clade L compared to clade S from which it had rapidly evolved early in the pandemic. Together, our findings support the previous hypothesis of Brufsky about possible ongoing competition between viral clades of varying virulence during the current pandemic^24^.

Our analysis of genomes metadata showed higher prevalence of severe or deceased cases among male patients than females in a statistically significant manner. The worse disease outcome of male patients was also reported by others^15–18^. Several assumptions have been made by scientists to justify this gender bias. Among them are female’s superior immune response^29^ and higher angiotensin converting enzyme type 2 (ACE2) activity in male or ovariectomized animal models^30^. ACE2 is the main receptor for SARS-CoV-2 spike through which it attaches to target cells^31^. Wambier and colleagues assumed androgen receptor genetic variation as a likely reason^32^. The receptor is thought to regulate transcription of the transmembrane protease serine 2 (TMPRSS2), responsible for S protein priming that allows viral fusion to host cell membranes^31^. To explain the role of the genomic variation of SARS-CoV-2 in the gender-biased COVID-19 outcome, the distribution of SARS-CoV-2 clades in viral genomes from male versus female patients was analyzed. Gender bias was evident for some clades. Strikingly, the clades GR and GV were found to be more significantly more prevalent in female patients than males. The least susceptible gender group are thus found to be show higher susceptibility to the newer SARS-CoV-2 clades.

Consistent with previous reports^16,33–35^, our analysis showed that severe disease or death was significantly more prevalent in elderly than in adults and children. This was previously explained by existence of comorbidities, immune senescence^36^ and alterations in ACE2 receptors^37^. Mild disease in children was also reported by many studies^15,38^. Contributing factors may include lower maturity and function of ACE2 receptors^39^ and viral co-infection that leads to limited replication of SARS-CoV-2 in the respiratory tract^40^. Interestingly, clade GV showed the highest prevalence among children compared to other age groups. Being the last to emerge among all clades, this further supports the adaptation-driven evolution hypothesis where new clades become more infectious to the least susceptible age group.

## Conclusion

The current analysis provides a statistical evidence on an ongoing adaptation-driven SARS-CoV-2 evolution whose outcome is higher viral infectivity and/or virulence. This is suggested by the biased distribution of the newer clades in geographic regions from which higher number of cases and deaths as well as higher CFRs were reported. More frequent isolation of the newer clades from the least susceptible populations including females and children was also noted. Given that the newer clades are thought to have higher virulence (GR according to the current study) and/or infectivity (GV according to the current study), this suggests that further evolution of the virus may put such groups at higher risk for COVID-19 worse outcome. However, it is worth mentioning that a successful genome-based epidemiologic analysis is limited by the inadequate and imbalanced number of genomes deposited in open access databases. Some constraints in this respect are the lack of whole genome sequencing facilities and data sharing policies by some countries. Accordingly, an experimental evidence is required to confirm or role out our hypothesis. Future studies are also recommended to address the impact of climate and lock down strategies on COVID-19 epidemiology.

## Methods

### SARS-CoV-2 genomes metadata

Metadata of all SARS-CoV-2 genomes submitted to the GISAID database (https://www.gisaid.org/CoV2020/), were accessed in January 25, 2021 (n = 419,256). Only genomes of viruses isolated from humans and those for which genomic clades were specified (n = 408,493) were selected for analysis. Metadata of genomes included information on collection date, geographic location, patient gender, patient age, patient clinical status and viral genome clade. The genomes were submitted by labs from 146 countries around the world. The continent distribution of the genomes included in the current study was as follows: 6,008 from Africa, 27,473 from Asia, 262,934 from Europe, 88,574 from North America, 18,358 from Oceania, and 5,178 from South America. Genomic clades were inferred by GISAID database and defined according to its nomenclature system at the time of data collection outlined in (https://www.gisaid.org/references/statements-clarifications/clade-and-lineage-nomenclature-aids-in-genomic-epidemiology-of-active-hcov-19-viruses/).

For age-based comparisons, entries for which patient age are available (n = 95,848) were classified into three age groups including children (up to 18 years), adults (18–64 years) and elderly (65 years or more). Cases for which patients clinical status were clearly specified were grouped into asymptomatic or mild group and severe or deceased group.

### Disease epidemiology data

Data of the disease epidemiology including total number of cases and total number of deaths in different countries were obtained from COVID‐19 situation dashboard of the World Health Organization available at (https://covid19.who.int) accessed in January 25, 2021.

The calculated median number of cases in the countries from which SARS-CoV-2 genomes were submitted to the database was 107,841 while the median number of deaths was 1,532 and that of the CFR was 1.6%. Contributing countries were grouped into two groups according to the relation between the national values of each of the disease epidemiology parameters to the median.

### Statistical analyses

Categorical data were expressed as percentages, while the median was used to describe the central tendency of the non-normally distributed numerical data. Group comparisons were done using Mann–Whitney U-test for numerical data and Chi-square (χ^2^) or Fisher’s exact test for categorical data. All statistical analyses were performed using the Statistical Package for Social Sciences (SPSS) software version 20.0 (IBM Corp., Armonk, NY, USA). P-value of less than 0.05 (two-tailed) was considered to be statistically significant.

## Data Availability

The datasets used and analyzed during the current study are available from the corresponding author on reasonable request.
